# Carboxy-terminal domain of Cas differentially modulates *c-Jun* expression, DNA synthesis, and membrane ruffling induced by insulin, EGF, and IGF-1

**DOI:** 10.1080/19768354.2018.1447013

**Published:** 2018-03-07

**Authors:** Sun-Ju Yi, Seong Yun Hwang, Myung-ju Oh, Kyunghwan Kim, Byung H. Jhun

**Affiliations:** a School of Biological Sciences, College of Natural Sciences, Chungbuk National University, Cheongju, Republic of Korea; b Department of Cogno-Mechatronics Engineering, Pusan National University, Busan, Republic of Korea

**Keywords:** Cas, microinjection, EGF, insulin, IGF-1, c-Jun expression, DNA synthesis, membrane ruffling

## Abstract

p130 Crk-associated substrate (Cas) is an adaptor protein associating with many other signaling proteins and regulates a various biological processes including cell adhesion, migration, and growth factor stimulation. However, the exact functional role of Cas in growth factor signaling pathway was poorly understood. Here we investigated the role of Cas and its domains in the effects of insulin, EGF, and IGF-1 on c-Jun gene expression, DNA synthesis, cytoskeletal reorganization. We found that microinjection of anti-Cas antibody and C-terminal domain of Cas (Cas-CT) specifically inhibited EGF-induced, but not insulin- or IGF-1-induced, c-Jun expression. Cell cycle progression and cytoskeleton reorganization induced by insulin and EGF, but not by IGF-1, were inhibited by microinjected anti-Cas and Cas-CT. In contrast, microinjection of the substate domain (Cas-SD) of Cas did not have any inhibitory effects. These results revealed that the Cas-CT is differentially implicated in insulin and EGF-mediated, but not IGF-1-mediated, c-Jun expression, DNA synthesis and membrane ruffling.

## 1. Introduction

Cas was originally identified as a major tyrosine-phosphorylated substrate in cells expressing v-Crk or v-Src oncoproteins (Sakai et al. 1994) and is now considered to be an adaptor protein which regulates a variety of biological processes, including cell adhesion, cell migration, growth factor stimulation, cytokine receptor engagement, and bacterial infection (O’Neill et al. 2000; Barrett et al. 2013; Nikonova et al. 2014). Recent genetic studies have indicated that Cas plays a critical role in the organization of the actin cytoskeleton. Cas-deficient mouse embryos die *in utero* and show marked cardiovascular defects which are linked to altered myofibril organization (Honda et al. 1998). Moreover, Cas-deficient fibroblasts show resistance to Src-induced transformation. Co-expression of v-Src and Cas led to a three-fold increase in the transcriptional activation of serum response elements (SREs) over the level induced by v-Src alone, suggesting that Cas plays a role in SRE activation and transformation by Src (Hakak and Martin 1999). This Cas-dependent activation of the SREs is dependent on the the carboxy-terminal domain of Cas (Cas-CT), a region that also requires extensive tyrosine phosphorylation in cells expressing activated v-Src.

In the basal state, tyrosine phosphorylation of Cas is increased by various extracellular stimuli, including growth factors such as epidermal growth factor (EGF), insulin-like-growth factor-1 (IGF-1), platelet-derived growth factor, and nerve growth factor (NGF) (Ribon and Saltiel 1996; Casamassima and Rozengurt 1997; Ojaniemi and Vuori 1997), B cell receptor engagement(Ingham RJ et al), and integrin-mediated cell adhesion (Petruzzelli et al. 1996). In contrast, Cas is dephosphorylated by insulin(Sorokin and Reed 1998), suggesting that Cas may be differentially regulated by insulin and other growth factors such as EGF and IGF-1.

Molecular cloning of Cas has revealed that it contains an amino-terminal SH3 domain, a central substrate domain composed of a cluster of SH2-binding sites, and a C-terminal domain. Each Cas domain has been shown to directly interact with a different set of signaling proteins. The SH3 domain associates with FAK, PTP1B, PTP-PEST, C3G, and CMS (Polte and Hanks 1995; Liu et al. 1996; Garton et al. 1997; Kirsch et al. 1998; Kirsch et al. 1999). The central SH2-binding substrate domain is highly tyrosine-phosphorylated and possesses docking sites for SH2 domain-containing proteins such as Crk, Nck, the p85 subunit of PI3-kinase, Grb2, phospholipase C-γ, and the protein tyrosine phosphatase Shp-2(Vuori et al. 1996; Manie et al. 1997; Schlaepfer et al. 1997). Finally, the Cas-CT interacts with c-Src, 14-3-3, BCAR3, and Chat/SHEP1 (Nakamoto et al. 1996; Cai et al. 1999; Garcia-Guzman et al. 1999; Sakakibara and Hattori 2000). Although these numerous interacting partners suggest that Cas plays an important role in the coordination and control of different cellular processes, the functional role of each Cas domain is not completely understood.

In the present study, we investigate the functional role of Cas and its domains in the effects of insulin, EGF, and IGF-1 on c-Jun gene expression, DNA synthesis, and cytoskeletal reorganization through single-cell microinjection. Our results show that Cas, particularly its C-terminal domain, is differentially involved in insulin- and EGF-induced, but not IGF-1-induced, c-Jun expression, DNA synthesis, and membrane ruffling.

## 2. Materials and methods

### 2.1. Cells culture and antibodies

Rat-1 fibroblasts overexpressing wild-type human insulin receptors (HIRc) (generous gift from Dr. Jerrold M. Olefsky, University of California, Sna Diego, CA) and COS-7 cells were maintained as described previously (Jhun et al. 1994). Anti-Cas antibody (C-20) was obtained from Santa Cruz. Rat anti-bromodeoxyuridine (BrdU) antibody was purchased from Accurate Chemical & Scientific Co. c-Jun antibody was obtained from BD biosciences. Rabbit anti-hemagglutinin (HA) antibody was from Upstate Biotechnology Inc. Anti-mouse IgG and -rabbit antibodies conjugated with fluorescein isothiocyanate (FITC) or tetrametyl rhodamine isothiocyante (TRITC) were obtained from Jackson Laboratories. TRITC-conjugated phalloidin were purchased from Sigma.

### 2.2. Plasmids construction, transfection and immunodetection

The genes encoding Cas full-length (FL) (residues 1 to 874) or Cas–CT (residues 668 to 874) or Cas-SD (residues 233 to 667) were prepared by PCR amplification and subcloned into pEGFP. COS-7 cells were cultured in 60 mm dishes containing acid-washed glass coverslips for 24 h and transiently transfected with 1.5 μg of pEGFP-Cas-FL, or pEGFP-Cas-CT, or pEGFP-Cas-SD for 3 h using Superfect (Qiagen, Valencia, CA) according to the manufacture’s protocol. 24 h after transfection, the cells were fixed with 3.7% formaldehyde and then permeabilized with 0.3% Triton X-100. For immunostaining of focal adhesions, anti-vinculin antibody (Sigma) and TRITC-conjugated anti-rabbit IgG antibody were used. The image was photographed with confocal microscope.

### 2.3. Single cell microinjection and immunostaining

Single cell microinjection was performed as described previously with some modifications (Yi et al. 2017). Briefly, HIRc cells were grown on acid-washed coverslips for 24 h and rendered quiescent by starvation for 24 h in serum-free DMEM. For nuclear injection, pcDNA-HA, pcDNA-HA-Cas-CT, or pcDNA-HA-Cas-SD was diluted in the nuclear injection buffer (50 mM HEPES, pH 7.2, 100 mM KCl, 50 mM NaPO_4_) with final concentration of 10 ng/ml. For cytosolic injection, anti-Cas antibody or GST-Crk-SH2 was diluted in PBS with final concentration of 2 mg/ml or 8 mg/ml, respectively. To detect c-Jun protein expression, microinjected cells were stabilized for 16 h followed by stimulation of either insulin (100 ng/ml), EGF (20 ng/ml), or IGF-1 (20 ng/ml) for 2 h. The immunostaining of c-Jun protein was performed as described previously (Na et al. 1999). For DNA synthesis, the cells were stabilized for 4 h after microinjection, and then stimulated with either insulin (100 ng/ml), EGF (20 ng/ml), or IGF-1 (20 ng/ml) for 24 h in the presence of BrdU. DNA synthesis was monitored as described previously(Jhun et al. 1994). For membrane ruffling, the microinjected cells were stabilized for 16 h, and then stimulated with insulin (100 ng/ml) for 10 min. The cells were fixed with 3.7% formaldehyde in PBS for 10 min, and then permeabilized with 0.3% Triton X-100 in PBS for 10 min. Then the injected cells were sequentially incubated with with TRITC-conjugated phalloidin (0.1 μg/ml) for 1 h at 37°C.

## 3. Results

### 3.1. C-terminal domain of Cas mediates c-Jun expression induced by EGF, but not by insulin or IGF-1

**Figure 1.**
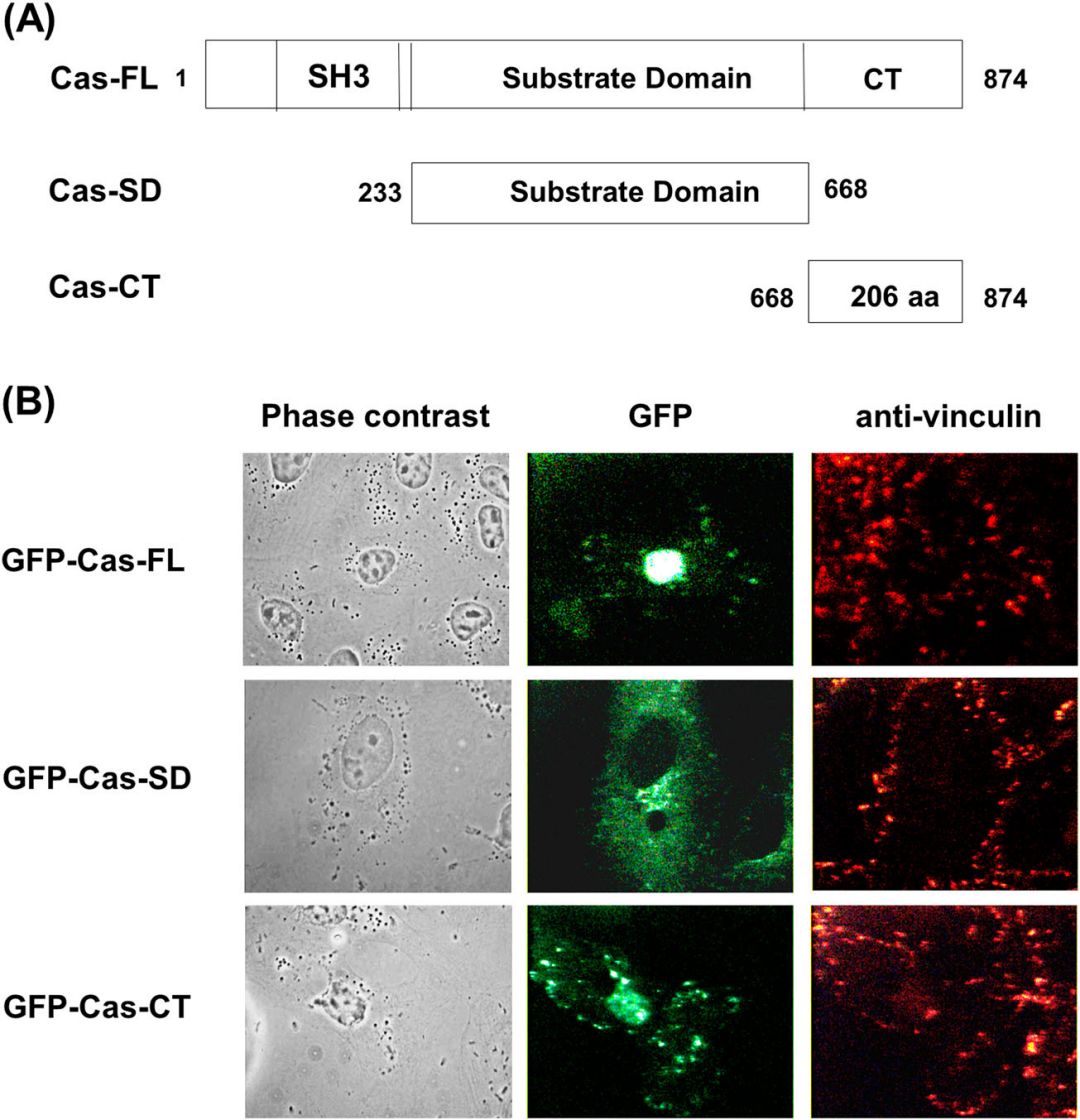
Schematic representation of Cas domain structure and cellular localization. (A) Deletion mutants of Cas. Carboxy-terminal domain of Cas (residues 668–874, CT) and substrate-binding domain of Cas (residues 233–667, SD) without FAK-binding SH3 domain and C-terminal domain of Cas, as depicted. (B) COS-7 cells were transfected with pEGFP- Cas-FL, or -CT, or –SD for 24 h and then stained with anti-vinculin antibody to visualize focal adhesions.

To understand the functional roles of Cas and its domains, we first constructed GFP-tagged expression vectors containing the Cas FL, the Cas-CT and the Cas-SD (Figure 1(A)). After transient transfection of COS-7 cells with GFP-Cas-FL or -CT or -SD, their subcellular localization was examined by confocal microscopic analysis. Cas-SD was widely distributed throughout the cytoplasm but was excluded from focal adhesion sites. In contrast, both the Cas-FL and the Cas-CT specifically localized to focal adhesion sites (Figure 1(B)). These results are consistent with previous studies that showed that the Cas-CT contains focal adhesion targeting sequences, suggesting that the Cas-CT may play a key role in focal adhesion dynamics by integrating various extracellular stimuli.

**Figure 2.**
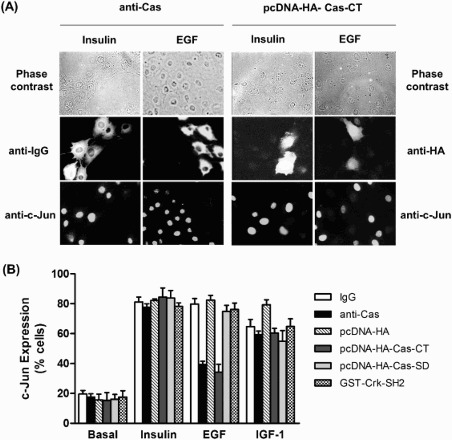
Effects of microinjection of anti-Cas and plasmid expressing Cas-CT or -SD on insulin, EGF and IGF-1 induced c-Jun Expression in HIRc cells. (A and B) Serum starved HIRc cells were microinjected with anti-Cas antibody (2 mg/ml), GST-Crk-SH2 (8 mg/ml), and pcDNA-HA-Cas-CT or -SD (10 ng/ml). After stabilization for 2 h (antibody and protein injection) or 16 h (plasmid injection), the cells were stimulated with insulin (100 ng/ml), EGF (20 ng/ml) or IGF-1 (20 ng/ml) for 4 h at 37°C. c-Jun expression in the injected cells was determined as described under ‘Materials and methods’. Bars represent results of total 1200 injected cells.

Since EGF, IGF-1, and insulin are well-known growth factors for c-Jun expression, and FAK-Src-Cas complexes are involved in activating the JNK (Jun N-terminal kinase) pathway (Dolfi et al. 1998), we next examined a possible role of Cas and its domains in the signaling pathway of insulin, EGF, and IGF-1 leading to c-Jun expression. To do this, we employed single-cell microinjection. First, an affinity-purified anti-Cas antibody with neutralizing activity of endogenous Cas was introduced into HIRc cells followed by stimulation with insulin, EGF, or IGF-1. c-Jun protein expression was monitored in the injected cells by indirect immunofluorescence staining using anti-c-Jun antibody. As shown in Figure 2, microinjection of the anti-Cas antibody significantly inhibited EGF-stimulated c-Jun expression. In contrast, microinjection of a control rabbit IgG did not have any inhibitory effect on c-Jun induction. Interestingly, c-Jun induction by insulin and IGF-1 was not affected by the anti-Cas antibody. These results strongly demonstrate that Cas is a pathway-specific regulator in the induction of c-Jun expression by EGF signaling, but not by insulin or IGF-1 signaling. To further determine which domain of Cas is involved in EGF-induced c-Jun expression, plasmids encoding HA-tagged Cas-CT or -SD were injected into the HIRc cells, and then c-Jun expression was analyzed by immunostaining with anti-c-Jun antibody following EGF, insulin, or IGF-1 treatment. As expected, the Cas-CT severely blocked EGF-induced c-Jun expression, whereas the Cas-SD failed to induce c-Jun expression mediated by EGF. Since the Cas-SD substrate domain contains putative SH2-binding motifs to which the Crk-SH2 domain preferentially binds, leading to activation of JNK via C3G and R-Ras (Dolfi et al. 1998; Mochizuki et al. 2000), the binding of Cas and Crk via the Crk SH2 domain might affect EGF-mediated c-Jun expression. To prove this, we microinjected GST-Crk SH2 into HIRc cells and then measured EGF-induced c-Jun expression levels. Consistent with microinjection of Cas-SD, GST-Crk SH2 protein did not abolish c-Jun expression. These results strongly indicate that the Cas-CT plays an important role in EGF-mediated c-Jun induction.

### 3.2. C-terminal domain of Cas regulates DNA synthesis induced by insulin and EGF, but not by IGF-1

**Figure 3.**
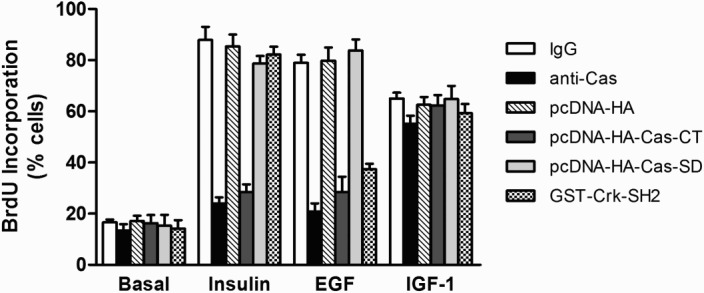
Effect of microinjected anti-Cas and plasmid expressing Cas-CT or -SD on insulin, EGF and IGF-1 induced DNA synthesis in HIRc-B cells. Serum starved cells were microinjected with anti-Cas antibody (2 mg/ml), GST-Crk-SH2 (8 mg/ml), pcDNA-HA-Cas-CT or –Cas-SD (10 ng/ml) as in Figure 2. After stabilization for 1 h (cytosol injection) or 4 h (nuclear injection), the cells were stimulated with insulin (100 ng/ml), EGF (20 ng/ml), and IGF-1 (20 ng/ml) for 16 h. To investigate DNA synthesis, the cells were further incubated with BrdU for 16 h, and then were fixed. DNA synthesis in the injected cells was determined as described in ‘Materials and methods’.

To further examine the functional roles of both the C-terminal domain and the substrate domain of Cas in insulin-, EGF-, or IGF-1-induced DNA synthesis, we again employed single-cell microinjection. Following stimulation, BrdU incorporation into newly synthesized DNA was monitored by indirect immunofluorescence staining. Figure 3 shows that the basal rates of DNA synthesis were unaffected by microinjected materials, indicating no harmful action of microinjection on the cells. Moreover, control rabbit IgG or mock plasmid had no adverse effects on DNA synthesis induced by insulin, EGF, or IGF-1. In contrast, microinjection of anti-Cas antibody or Cas-CT plasmid inhibited EGF- and insulin-stimulated BrdU labeling by 60% ∼ 70%. Interestingly, the injected anti-Cas antibody or Cas-CT plasmid did not suppress IGF-1-stimulated DNA synthesis. Moreover, microinjected Cas-SD plasmid did not inhibit DNA synthesis induced by insulin, EGF, or IGF-1. We also examined the functional roles of Crk-SH2 protein in insulin-, EGF-, or IGF-1-induced DNA synthesis. Microinjection of GST-Crk-SH2 inhibited DNA synthesis induced by EGF (40% inhibition) but not by insulin or IGF-1. These results suggest that the Cas-CT is necessary for insulin and EGF to exert their mitogenic stimulatory effects, but not for IGF-1.

### 3.3. C-terminal domain of Cas mediates membrane ruffling induced by insulin

**Figure 4.**
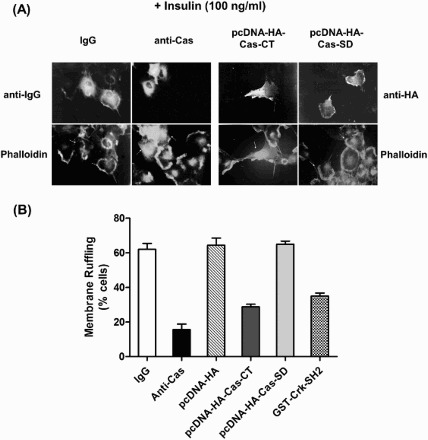
C-terminal domain of Cas regulates insulin-induced membrane ruffling. (A and B) Serum starved cells were microinjected with anti-Cas antibody (2 mg/ml), GST-Crk-SH2 (8 mg/ml), pcDNA-HA-Cas-CT or -Cas-SD (10 ng/ml) as in Figure 2. After stabilization for 1 h (antibody or protein injection) or 16 h (plasmid injection), the cells were stimulated with the insulin at concentration of 100 ng/ml for 10 min at 37°C. Membrane ruffling was monitored with phalloidin staining.

Next, we examined whether the Cas-CT and the Cas-SD are involved in the membrane ruffling induced by insulin and EGF. For insulin-mediated membrane ruffling, we used HIRc cells. After microinjection of anti-Cas antibody or plasmids expressing Cas-CT or –SD, cells were then stimulated with insulin (100 ng/ml). Membrane ruffling was visualized by immunostaining with TRITC-conjugated phalloidin. As shown in Figure 4, microinjection of the anti-Cas antibody or the plasmid encoding Cas-CT clearly inhibited insulin-induced membrane ruffling. However, microinjection of a Cas-SD-expressing plasmid did not have any effect on membrane ruffling. Consistent with the results for the insulin signaling pathway, the Cas-CT inhibited EGF-induced membrane ruffling in COS-7 cells (data not shown). Microinjection of GST-Crk-SH2 protein inhibited membrane ruffling induced by insulin or EGF. These results strongly indicate that the Cas-CT plays an important role in the signal transduction pathways of insulin and EGF that lead to cytoskeleton reorganization.

## 4. Discussion

A growing body of evidence suggests that Cas is involved in multiple biological processes, including migration, cell cycle control, and apoptosis (Barrett et al. 2013). Cas contains 15 potential tyrosine phosphorylation sites which can serve as SH2-binding motifs. Nine of these sites conform to the SH2-binding motif for Crk. Complexes of Cas and downstream effectors are known to lead to the activation of signaling pathways controlling cellular functions such as cytoskeleton organization (Altun-Gultekin et al. 1998), cell migration (Klemke et al. 1998), and JNK activation (Dolfi et al. 1998). However, the exact role of Cas has not been identified. In the present study, we demonstrate that the Cas-CT plays an important role in insulin- and EGF- induced cytoskeletal organization of membrane ruffling, DNA synthesis, and c-Jun gene expression.

The Cas-CT has been known to interact with several proteins such as Src, 14-3-3, Caspase-3, and BCAR3. For example, 14-3-3 proteins participate in integrin-activated signaling pathways through interaction with Cas (Garcia-Guzman et al. 1999). The interaction of Cas with Src promotes tamoxifen resistance of breast cancer cells through the EGF signaling pathway (Riggins et al. 2006). Recently, it has been reported that the Cas-BCAR3 complex led to Src activation and cell migration (Riggins et al. 2003). In this study, we observed that overexpression of the Cas-CT differentially inhibited the signaling pathways of insulin, EGF, and IGF-1. DNA synthesis and membrane ruffling induced by both insulin and EGF were blocked by Cas-CT. Moreover, Cas-CT also blocked EGF-induced, but not insulin- or IGF-1-induced, c-Jun expression. Based on our observations, together with previous studies, it is possible that EGF and insulin signaling pathways adopt distinct Cas-downstream effector complexes to induce c-Jun expression. Therefore, further studies of the various roles of Cas-effector complexes in the signaling of different growth factors are required.

It was previously reported that Cas plays an important role in regulating cell motility through its associations with FAK and Crk (Polte and Hanks 1995). One possible mechanism of Cas-mediated cell migration is through the Rho family of GTPases since Crk has been suggested to mediate some of its cellular morphological effects through activation of Rho signaling pathways, which are known to regulate specific actin cytoskeletal functions. Moreover, the major downstream effectors of Crk activate the JNK family of MAP kinases, leading to cell cycle progression (Tanaka et al. 1997). Consistent with previous studies (Nakashima et al. 1999), we also observed that microinjection of the SH2 domain of Crk inhibited EGF-induced DNA synthesis. Interestingly, however, microinjection of the Crk-SH2 domain did not inhibit DNA synthesis induced by insulin. These findings raise the possibility that the function of Cas in mitogenesis does not fully depend on the Crk-mediated pathway and that another domain or other downstream effectors of Cas may regulate insulin-induced mitogenesis. Further studies are required to identify the function of each Cas domain and the roles of these regions in mitogenesis and cytoskeletal reorganization.

An unsolved question in our study is the role of Cas-SD in the growth factor signaling pathways linking to c-Jun, cell cycle progression, and membrane architecture. Since the substrate domain functions as a docking domain and contains 15 repeats of the YxxP consensus motif, which are mainly responsible for association with Crk, we expected that the growth factor signaling processes would be affected by the introduction of the Cas-SD. However, we observed that the Cas-SD, unlike Crk-SH2, had no effect. One explanation for this observation is that the Cas-SD alone could not localize to the focal adhesion sites, large multiprotein assemblies through which mechanical forces and regulatory signals are transmitted. A further possibility is that Cas-CT or -SH3 domain may compensate for the lack of the substrate domain of Cas. Future studies are needed to elucidate these questions.
